# Errors in implant orientation estimation in novice vs. experienced surgeons during reverse shoulder arthroplasty for a superior glenoid wear pattern

**DOI:** 10.1016/j.jseint.2024.12.008

**Published:** 2025-01-15

**Authors:** Ryan Lohre, Aaron J. Bois, Akshay Lobo, J Whitcomb Pollock, Peter Lapner, George S. Athwal, Danny Goel

**Affiliations:** a Department of Orthopaedic Surgery, Harvard Medical School, Massachusetts General Hospital, Boston Shoulder Institute, Boston, MA, USA; b Section of Orthopaedic Surgery, Department of Surgery, University of Calgary, Calgary, AB, Canada; c McCaig Institute for Bone and Joint Health, Cumming School of Medicine, University of Calgary, Calgary, AB, Canada; d Department of Orthopaedics, University of British Columbia, Vancouver, BC, Canada; e Division of Orthopaedic Surgery, Ottawa Hospital Research Institute; University of Ottawa, Ottawa, ON, Canada; f Roth McFarlane Hand and Upper Limb Center, Western University Schulich School of Medicine and Dentistry, London, ON, Canada

**Keywords:** Reverse shoulder arthroplasty, Surgical training, Kinesthetic learning, 3D Computed tomography, Digitally reconstructed radiograph, Resident training

## Abstract

**Background:**

Glenoid baseplate orientation in reverse shoulder arthroplasty influences clinical outcomes, complications, and failure rates. This study aimed to determine novice and experienced shoulder surgeon’s ability to accurately characterize glenoid component orientation in an intraoperative scenario.

**Methods:**

Glenoid baseplates were implanted in 8 fresh frozen cadavers by novice surgical trainees. Glenoid baseplate version, inclination, augment rotation, and superior-inferior center of rotation offset were then measured using in-person visual assessments by novice and experienced shoulder surgeons immediately after implantation. Glenoid orientation parameters were then measured using 3-dimensional (3D) computed tomography (CT) scans with digitally reconstructed radiographs (DRRs) by 2 independent observers with a 1-month time interval between repeat measurements. Bland-Altman plots were produced to determine the accuracy of glenoid orientation using standard intraoperative assessment compared to postoperative 3D CT scan results. Interclass correlation coefficients were produced for measurements, rated as 0.01-0.39 poor, 0.40-0.59 fair, 0.60-0.74 good, and 0.75-1.00 excellent.

**Results:**

Visual assessment of glenoid baseplate orientation showed “poor” to “fair” correlation to 3D CT DRR measurements for both novice and experienced surgeon groups for all measured parameters. There was a large discrepancy between intraoperative visual assessments and 3D CT DRR measurements for all parameters. Errors in visual assessment of up to 20° of inclination by experienced surgeons (*P* = .03), and 8 mm supero-inferior center of rotation offset by novice surgeons (*P* = .50) occurred. Experienced surgeons had greater measurement error than novices for all measured parameters.

**Conclusion:**

Intraoperative measurement errors in glenoid placement are present for both inexperienced and experienced surgeons. Kinesthetic input during implantation may improve orientation understanding.

The original indication for reverse total shoulder arthroplasty was to manage the complex problem of rotator cuff tear arthropathy (CTA) and currently remains its primary indication. Recent studies of reverse shoulder arthroplasty (RSA) show reliable improvements in patient outcomes with small, well-characterized complication profiles.^36^^,^^37^ Proximal migration of the humeral head in patients with CTA produces glenoid wear patterns as previously characterized by Favard.^38^ With prolonged posterosuperior rotator cuff deficiency, glenoid erosion progresses from central and concentric (E1) to eccentric patterns including posterosuperior (E2 and E3) to inferior (E4) bone loss.^38^ Correcting these deformities has been accomplished through selective “high side” reaming, bone grafting, and augmented glenoid baseplates.^24^ Failure to recognize and correct for these patterns can lead to incorrect superior-inferior tilt or offset of the glenoid baseplate. A superiorly inclined baseplate (and therefore glenosphere), is a risk factor for both instability and notching, while superior center of rotation (COR) offset is a risk factor for scapular notching.^2^ Both complications increase revision rates and have been shown to decrease patient-reported outcome measures.^7^^,^^12^^,^^17^ Augmented glenoid baseplates have the potential advantage of preserving glenoid bone stock by minimizing reaming and avoiding potential graft resorption and premature component loosening and failure. Despite availability of augmented RSA baseplates to surgeons, clinical data and outcomes remain limited. Favard E1-type wear patterns may predispose to erroneous implant placement; however, this has not been studied in other superior wear patterns of CTA.^3^

Reports of learning curves in RSA are variable though demonstrate significantly more complications in the early period.^8^^,^^19^^,^^23^ Shoulder surgical volume has also been shown to be an individual risk factor for reoperation rates and outcomes in short-term and long-term follow-up.^4^^,^^32^ Data from the most recent American Academy of Orthopaedic Surgeons census document reveal that the majority of surgeons are performing less than 20 shoulder arthroplasties per year, and considered low-volume.^1^ Hands-on, kinesthetic input has been shown to be particularly important in learning alongside visual cues.^13^^,^^31^^,^^33^ Shoulder arthroplasty provides a unique opportunity to evaluate the importance of visual and kinesthetic cues on implant placement because of limited exposure of the scapula.^28^

The purpose of this study was to determine the accuracy and reliability of intraoperative visual assessment for the placement of augmented glenoid baseplates by both novice and experienced surgeons. The primary objective was to compare intraoperative visual assessment of baseplate orientation to 3-dimensional (3D) computed tomography (CT) scan measurements for novice and experienced surgeons. Secondary objectives were to determine if errors in measurement reach clinically significant thresholds. Our hypothesis is that implantation and measurement errors will be present for intraoperative visual assessment of baseplate orientation.

## Materials and methods

### Cadaveric specimen preparation and baseplate insertion

Eight fresh-frozen upper limb cadaveric specimens (ie, scapula to hand) were prepared using a standard deltopectoral approach per a previously published protocol.^27^ There was no appreciable initial glenoid pathology evident. A superior glenoid wear pattern (ie, Favard E2) was created using a custom jig by 2 of the senior authors (A. J. B. and J. P.).^27^ Eight postgraduate year 4-5 residents attending the Canadian Shoulder and Elbow Society (CSES) Residents and Fellows course were enrolled following ethics approval by the University of Ottawa. Instruction was provided to the residents through teaching sessions on RSA baseplate insertion including recommended insertion parameters (neutral version, neutral to 10° of inferior inclination, neutral rotation such that the superior and inferior screw holes align with the vertical axis of the glenoid vault, and neutral inferior offset such that the central fixation point of the glenoid baseplate aligns with the central axis of the vault and the inferior most aspect of the baseplate aligns with the margin of the inferior glenoid without overhang). The residents performed an RSA using an augmented glenoid baseplate (Zimmer Comprehensive Reverse Shoulder Arthroplasty system; Zimmer, Warsaw, IN, USA) on randomly selected cadavers. Following glenoid baseplate insertion, novices and 3 experienced surgeons (A. J. B., P. L., and J. P.) were asked to describe the implanted glenoid baseplate orientation parameters in terms of glenoid version (degrees), inclination (degrees), rotation (clock face), and superior/inferior offset from the ideal center point (millimeters). This was produced with visual inspection alone without measurement devices to replicate intraoperative surgeon assessments. Experienced surgeons did not interact with (ie, observe) or instruct novices as to the correct placement of the glenoid. Novice surgeons were defined as current residents and experienced surgeons were defined as those fellowship trained in shoulder surgery and currently an attending in practice in shoulder and upper extremity surgery, regularly performing RSA. Both resident and experienced surgeons recorded their subjective interpretations separately to avoid bias. Measurements were compared to clinically acceptable limits of 0° to -10° retroversion, 0° to -10° inferior tilt, neutral rotation, and neutral inferior offset.^14^^,^^25^^,^^34^

### Radiographic measurements

Individual scapular specimens underwent a CT scan using a GE Medical Discovery CT750 (Chicago, IL, USA) thin slice (slice thickness: 0.625 mm; image matrix: 512 × 512 pixels; pixel spacing 0.488 mm) sequences in axial, coronal, and sagittal reformats. Digital Imaging and Communications in Medicine data were used to produce 3D reconstructions in 3D Slicer (www.slicer.org). The scapular plane was defined for each scapula using the inferior pole of the scapula (A), the center of the glenoid (B), and the trigonum scapulae (C). Based on this plane, a Cartesian (x, y, z) coordinate system was produced with the z-axis (A-B), x-axis normal to the ABC plane, and y-axis as the cross-product of the x-z axes.^40^ 3D digitally reconstructed radiographs (DRRs) were produced for all scapulae using 3D Slicer via volume rendering and ray casting to produce an overlay image similar to a clinical radiograph.^26^^,^^40^

**Figure 1:**
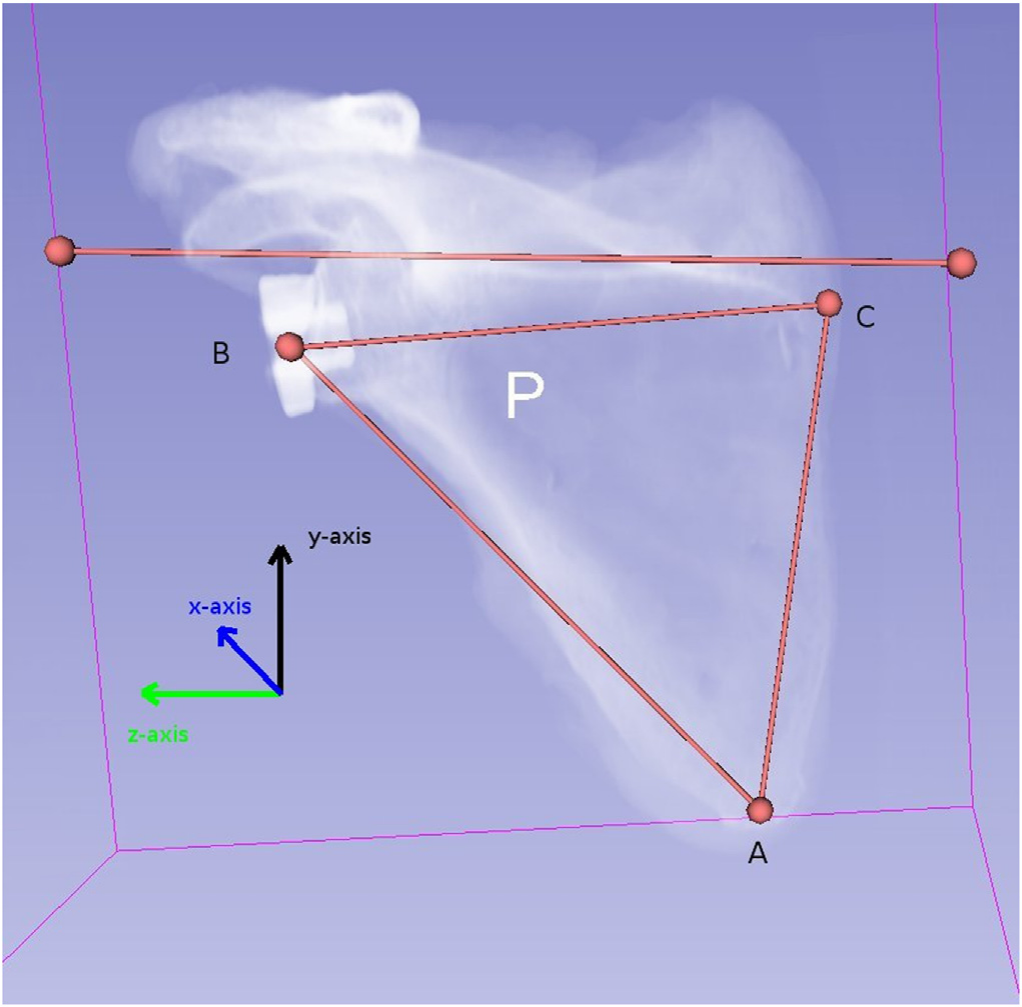
Representative digitally reconstructed radiograph (DRR) with Cartesian coordinates based on relevant landmarks creating the scapular plane.

Measurements were performed on 3D CT DRRs by 2 independent raters (R. L. and A. L.) with a 1-month time interval between replicate measures. The 3D CT DRRs were first visualized normal to the scapular plane (ABC). DRRs were adjusted for viewing perspective to produce images orthogonal to the glenoid baseplate in the y-axis (glenoid baseplate version) and z-axis (glenoid baseplate inclination and offset). Next, using the Cartesian coordinate system, the viewing angle parallel to the scapular plane was produced thus replicating a scapular-Y clinical radiograph. Viewing perspective adjustments in the x-axis produced orthogonal images to the glenoid baseplate to determine glenoid rotation. Segmentation of the volume renderings to exclude the baseplate resulted in images visualizing screw tracts in the prepared glenoid fossa to assist in determining screw perforation or placement in the spinoglenoid notch. DRRs were exported to Image J software (Bethesda, MD, USA) for measurements (Fig. 1).

### Statistical analysis

Statistical analyses were performed using R (R Foundation for Statistical Computing, Vienna, Austria). To determine measurement reproducibility, intraclass correlation coefficients and interclass correlation coefficients (ICCs) were produced for both in-person (subjective) measures and CT scan measurements. The mean difference, 95% confidence interval (CI), and related-samples Wilcoxon tests were performed. ICC values were rated as 0.01-0.39 poor, 0.40-0.59 fair, 0.60-0.74 good, and 0.75-1.00 excellent.^9^ The version, inclination, rotation, and offset mean differences were assessed between in-person and 3D CT DRRs, which were considered the true reference. Prior investigations have validated the use of DRRs compared to radiographs and 3D CT.^20^^,^^39^ The mean difference, 95% CI, ICC, and related-samples Wilcoxon test were again performed for each individual observer. Bland-Altman plots were produced for each measured value for graphical representation of mean difference. Bland-Altman plots were chosen as they provide information on the differences between measurements of the same item, not agreements, which can be misleading. The mean differences of measurements on these plots describe measurement bias. An overlapping agreement interval (negligible mean differences) indicated minimal bias between measurements. The y-axis consists of the difference between 2 measurement types plotted against the average of the 2 techniques (x-axis). Solid lines represent the mean difference and dashed lines represent the mean difference ± the double standard deviation (SD) of the differences, representing the 95% confidence of the range of errors. A priori reference values are required for interpretation of Bland-Altman plots, which were considered clinically acceptable limits of 0° to -10° retroversion, 0° to -10° inferiror tilt, neutral rotation, and neutral inferior offset.^14^^,^^25^^,^^34^ If measurements fall within the clinically accepted limits on a Bland-Altman plot, then this method of measurement may be used. In contrary, if measurements fall outside of these clinically acceptable limits on the Bland Altman plots, then the measurement modality is unacceptable in practice. Spearman correlation coefficients were calculated to detect statistical significance of any noted relationship between differences and magnitude of measurements.

## Results

### 3D CT measurements

**Figure 2:**
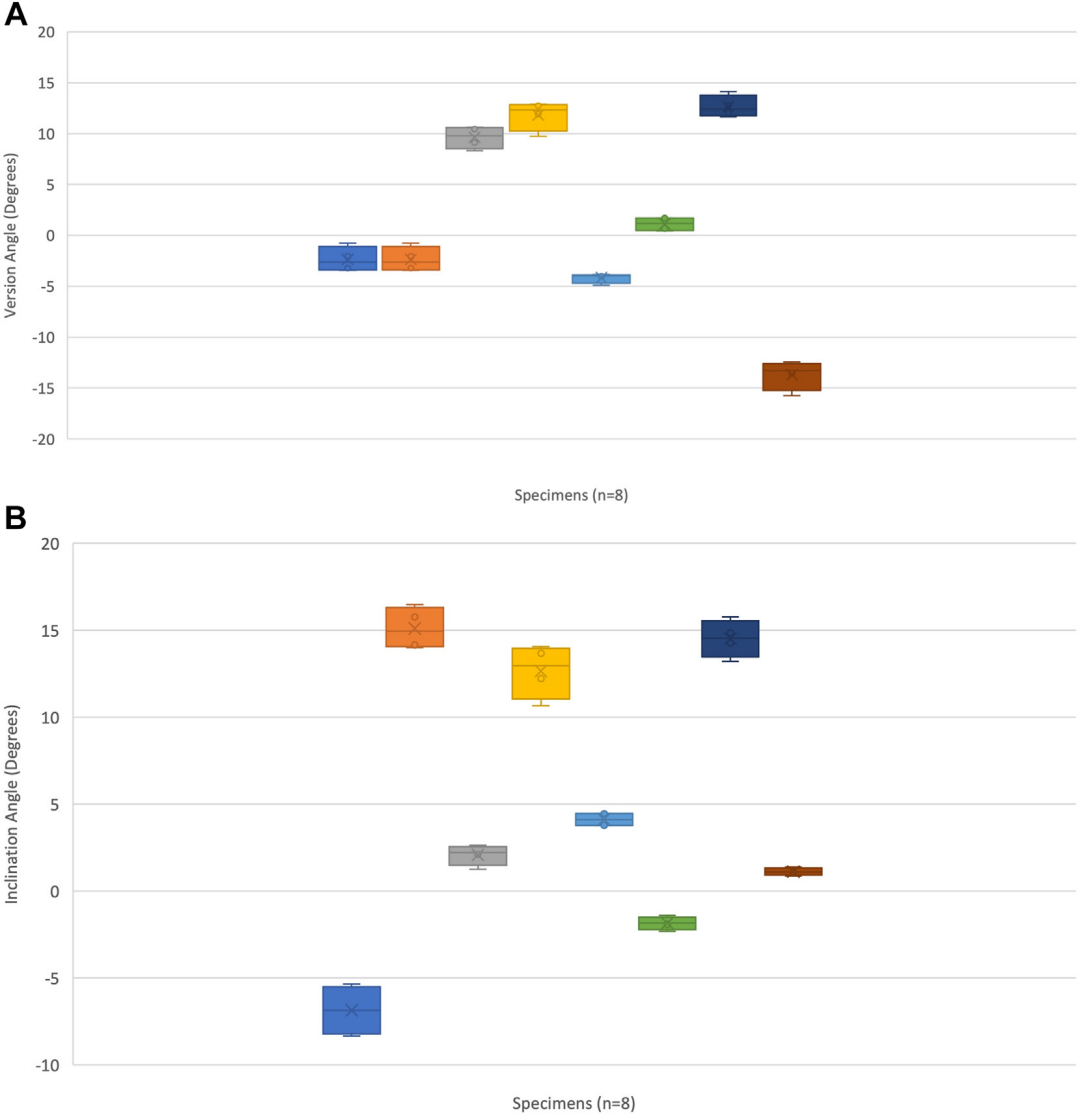

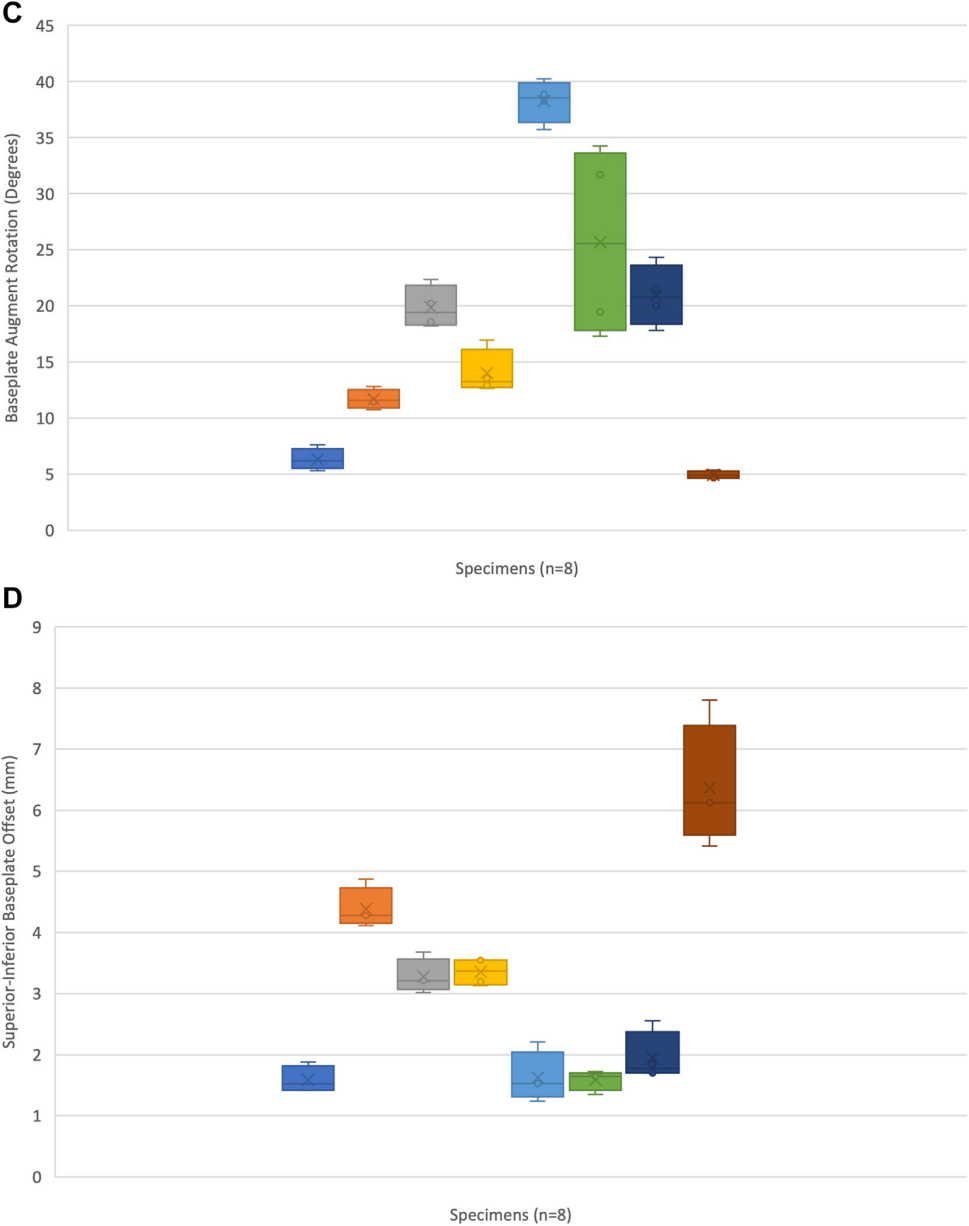
Tukey plots for (**A**) version, (**B**) inclination, (**C**) rotation, and (**D**) center of rotation (COR) offset. Dark horizontal lines are clinically relevant parameters including (**A**) neutral version, (**B**) −10° inferior tilt, (**C**) neutral rotation, and (**D**) neutral COR offset.

Demographic results are seen in Table I. The average augmented glenoid baseplate orientation measured from 3D CT was 2° anteverted (SD, 9°), 5° (SD, 8°) superior inclination, 17° (SD, 11°) malrotation, and 3.0 mm COR superior offset (SD, 1.8) (Table II). 3D CT DRRs showed an “excellent” measurement reliability of 0.97 (95% CI: 0.93-0.99). Reliability of measurements can be seen in Table III. These values were used as reference for further comparisons. Figure 2 shows Tukey plots of baseplate location for each measured parameter using 3D CT. Tukey plots illustrated that half (n = 4) of the residents placed the baseplate in anteversion, 2/3 (n = 6) were superiorly inclined, 2/3 (n = 6) were malrotated by >10°, and 100% (n = 8) were superiorly offset from the COR.

**Table I:** Participant demographics.

	Resident (n = 8)		
	Mean (SD)	Median (range)	Notes
Age (mean + SD)	32.7 (2.8)	33 (28-36)	
Gender	Male = 6 Female = 2 Undisclosed = 0		
Postgraduate training level	PGY4 = 3 PGY5 = 5		
Hand dominance	Left = 2 Right = 6		
Any corrected vision?	Yes = 6 No = 2		
Subjective experience with shoulder surgical approaches^1^	3.5 (0.9)	3.5 (2-5)	Likert scale 1-5 1: Not familiar 2: Not very familiar 3: Somewhat familiar 4: Familiar 5: Very familiar
Prior experience with RSA^1^	3.0 (0.5)	3.0 (2-4)	Likert scale 1-5 1: Not familiar 2: Not very familiar 3: Somewhat familiar 4: Familiar 5: Very familiar
Shoulder-specific surgical courses attended^11^	1.6 (0.9)	1 (1-3)	
Number of RSA completed acting as primary surgeon^2^	1.25 (0.4)	1 (1-2)	Likert scale 1-4 1: 0 cases 2: 1-10 cases 3: 10-20 cases 4: >20 cases

*SD*, standard deviation; *PGY*, postgraduate year; *RSA*, reverse shoulder arthroplasty.

**Table II:** Values for RSA using intraoperative and 3D CT DRR measurements.

	Version (degrees)	Inclination (degrees)	Rotation (degrees)	Offset (mm)
Intraoperative				
Novice (mean ± SD)	−2.25 ± 5.6	−1.75 ± 7.4	26.25 ± 20.7	2.8 ± 3.3
Experienced (mean ± SD)	−2.5 ± 6.3	−4.38 ± 15.5	28.12 ± 18.8	1.8 ± 2.4
Mean difference (Mann-Whitney U test)	−0.25 (*P* = .49)	−2.6 (*P* = .46)	1.8 (*P* = .41)	1.0 (*P* = .61)
3D CT DRR	2.0 ± 9.0	5.0 ± 8.0	17 ± 11	3.0 ± 1.8
Mean difference novice (intraoperative 3D CT) (Mann-Whitney U test)	−4.0 (*P* = .24)	−7.4 (*P* = .017)	6.4 (*P* = .39)	−0.2 (*P* = .50)
Mean difference experienced (intraoperative 3D CT) (Mann-Whitney U test)	−4.5 (*P* = .13)	−8.2 (*P* = .09)	10.7 (*P* = .24)	−1.2 (*P* = .24)

*RSA*, reverse shoulder arthroplasty; *3D*, 3-dimensional; *CT*, computed tomography; *DRR*, digitally reconstructed radiograph; *SD*, standard deviation.

**Table III:** Reliability measurements between participants (in-person) and radiographic observers.

Modality	Intraclass (ICC, 95% CI)	Interclass (ICC, 95% CI)
In-person assessment	-	0.84 (95% CI: 0.69-0.92)
Version (Friedman’s)	-	0.17 (95% CI: 0.72-0.79)
Inclination (β angle)	-	0.63 (95% CI: 0.22-0.92)
Augment rotation (degrees)	-	0.75 (95% CI: 0.15-0.95)
Superoinferior COR offset (mm)	-	0.17 (95% CI: 0.68-0.79)
3D CT DRR	0.99 (95% CI: 0.98-0.99)	0.95 (95% CI: 0.92-0.96)
Version (Friedman’s)	0.99 (95% CI: 0.98-0.99)	0.98 (95% CI: 0.94-0.96)
Inclination (β angle)	0.98 (95% CI: 0.93-0.99)	0.97 (95% CI: 0.93-0.99)
Augment rotation (degrees)	0.98 (95% CI: 0.90-0.99)	0.85 (95% CI: 0.62-0.94)
Superoinferior COR offset (mm)	0.97 (95% CI: 0.85-0.99)	0.89 (95% CI: 0.72-0.96)

*ICC*, interclass correlation coefficient; *CI*, confidence interval; *COR*, center of rotation; *3D*, 3-dimensional; *CT*, computed tomography; *DRR*, digitally reconstructed radiograph; *β angle*, represents the ‘global glenoid inclination’ and is the angle between the line of the glenoid fossa and the floor of the supraspinatus fossa.

### Novice group results

The mean intraoperative assessments by the novice surgeon group for version (−2.25° SD, 5.6° vs. 2.0° SD, 9.0; *P* = .11), component rotation (26.25° SD, 20.7° vs. 17.0° SD, 11.0; *P* = .24), and COR superior offset (2.8 mm SD, 3.3 mm vs. 3.0 mm SD, 1.8 mm; *P* = .92) were not significantly different from mean 3D CT values. Intraoperative inclination was significantly different (−1.75° SD, 7.4° vs. 5.0° SD, 8.0°; *P* = .0009). To better understand the measurement errors, the mean differences between intraoperative assessment and 3D CT measurements (Table II), and reliability of measures, were evaluated (Table III). The mean difference in inclination was significant (−7.4 95% CI −14.2 to −0.57; *P* = .017), while version (−4.0 95% CI: −18.1 to 10.1; *P* = .24), rotation (6.4 95% CI: 31.6-44.4; *P* = .39), and COR offset (−0.2 95% CI: −8.2 to 7.8; *P* = .50) were not. Measurement reliability for all parameters were “fair” to “poor” (Table III).

**Figure 3:**
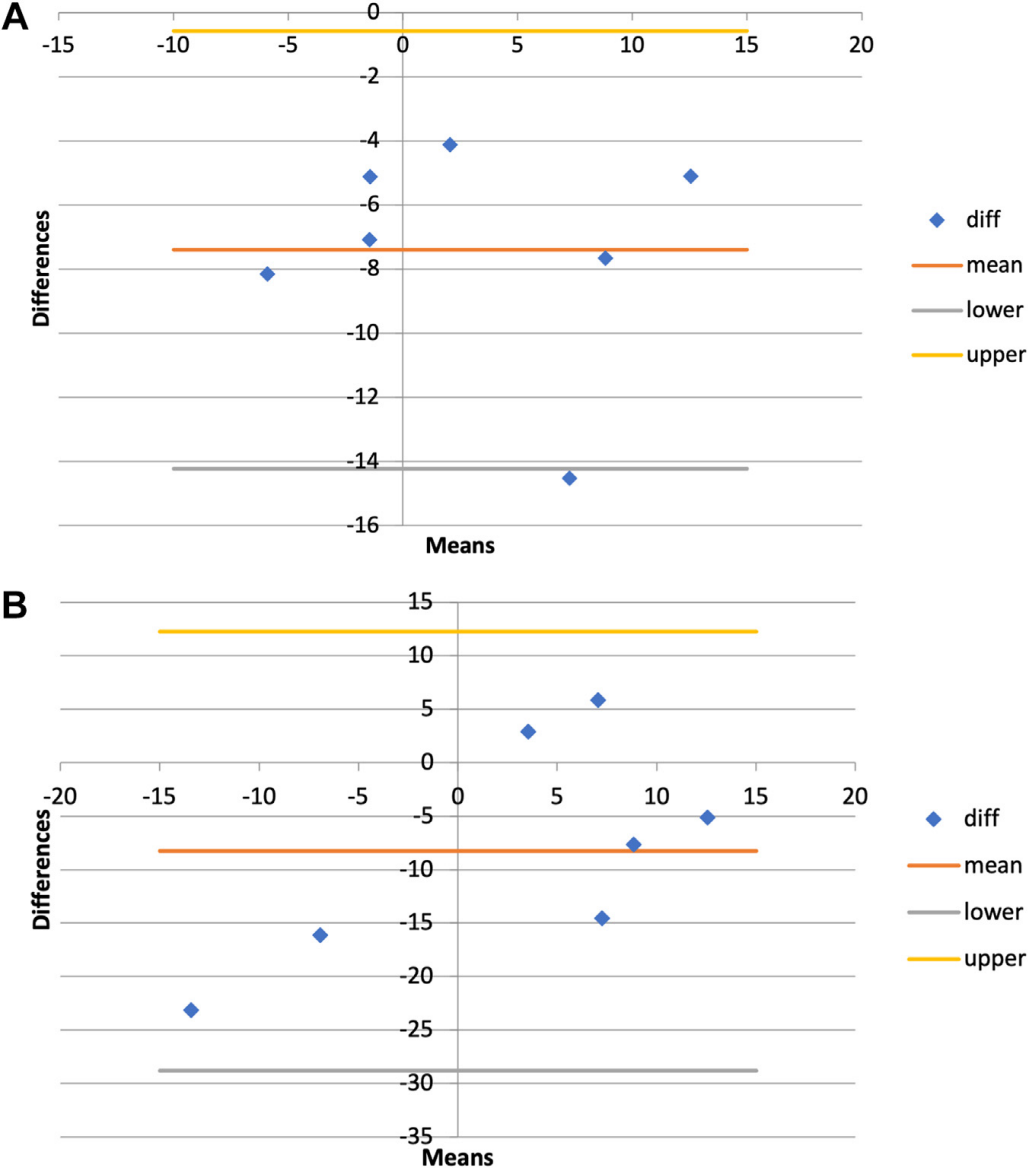
Representative Bland-Altman plots for (**A**) novice assessment and (**B**) experienced assessment of component inclination. The y-axis consists of the difference between 2 measurement types plotted against the average of the 2 techniques (x-axis). Solid lines represent the mean difference and dashed lines represent the mean difference ± the double standard deviation of the differences, representing the 95% confidence of the range of errors.

Bland-Altman plots provided measurement bias, as well as coefficients of repeatability and limits of measurement agreement. These values illustrate where intraoperative evaluators tended to think glenoid baseplates were oriented compared to the reference standard, as well as provide interpretation on how accurate repeat measurements would be by the same population and therefore the overall error associated with measurements (Table IV). Eighty-six percent of version assessments by the novice group will have an error of up to 13°. Similarly for inclination, 86% of intraoperative assessments have an error of up to 6°. One hundred percent of intraoperative rotational assessments will have an error of up to 36°, and 86% of offset measurements will have an error of up to 8 mm (Fig. 3).

**Table IV:** Comparison of in-person and 3D CT DRR.

	Clinical thresholds	3D CT DRR measurements of baseplate orientation	Novice assessment error	Experienced assessment error	Acceptability∗
Version	0°-10° retroversion	2° anteversion (SD, 9°)	13°	15°	Unacceptable
Inclination	0°-10° inferior	7° superior (SD, 8°)	6°	20°	Unacceptable
Rotation	Neutral	17° malrotation (SD, 11°)	36°	39°	Unacceptable
COR offset	Neutral	3.3 mm superior (SD, 1.8)	8 mm	4 mm	Unacceptable

*3D*, 3-dimensional; *CT*, computed tomography; *DRR*, digitally reconstructed radiograph; *SD*, standard deviation; *COR*, center of rotation.

∗ Acceptability of intraoperative glenoid orientation assessment by both novice and experienced surgeons determined by mean differences of assessments to 3D CT, reference, measurement bias, and error from Bland-Altman plots. Mean differences and error consistently outside of a-priori clinical thresholds.

### Experienced group results

The mean intraoperative assessments by the experienced surgeon group for version (−2.5°, SD 6.3° vs. 2.0° SD, 9.0; *P* = 17), rotation (28.12° SD, 18.8 vs. 17.0° SD, 11.0; *P* = .16), and COR offset (1.8 mm, SD, 2.4 mm vs. 3.0 mm SD, 1.8 mm; *P* = .13) were not significantly different from mean 3D CT values. Intraoperative inclination was significantly different (−4.38° SD, 15.5° vs. 5.0° SD, 8.0°; *P* = .03) (Table II). Mean differences were not statistically significant for version (−4.5 95% CI: −20.3 to 11.5; *P* = .13), inclination (−8.2 95% CI: −28.8 to 12.3; *P* = .09), rotation (10.7 95% CI: 29.5-50.9; *P* = .24), or COR offset (−1.2 95% CI: −5.6 to 3.2; *P* = .24) (Table II). Experienced surgeon measurement reliability was “fair” to “poor” and similar to the novice group. The experienced group showed greater mean differences compared to the novice surgeon group for all orientation parameters (Table II).

Experienced surgeon Bland-Altman plots showed similar, although greater, bias of measurement. Experienced surgeon measurement reliability compared to 3D CT DRR showed 100% of assessments had errors of up to 15° for version, 20° for inclination, 38° for rotation, and 4 mm for offset (Table IV). The experienced group overall had greater potential for error in assessment than the novice group.

## Discussion

This analysis demonstrates that both novice and experienced surgeons may incorrectly bias placement of augmented glenoids for RSA in Favard E2 wear patterns. Additionally, both novice and experienced surgeons may be inaccurate in their assessment of placement of augmented baseplates to levels of clinical significance.

Inclination and COR offset errors can result in instability and scapular notching. Although contemporary designs have decreased rates of early and late instability, it remains one of the most common reported complications of RSA (3.3%).^37^^,^^42^ Superior inclination is a risk factor for instability and notching, while inadequate inferior offset predisposes to scapular notching.^2^ Scapular notching has been shown to reduce functional range of motion and patient-reported outcome measures.^30^ Limits of agreement representing measurement accuracy in Bland-Altman plots are only important when considering a-priori clinically significant values.^16^ In our study, both novice and experienced surgeon in-person assessments of baseplate orientation fell outside of clinically acceptable parameters. Both groups incorrectly biased inclination and offset as inferior when in fact two-third of baseplates were superiorly tilted and 100% were superiorly offset in their COR. The large coefficient of repeatability (error) for novice (6°) and experienced surgeons (19°) measuring inclination indicates that both groups of surgeons may think they have correctly placed an augmented glenoid baseplate in clinically acceptable parameters when in fact, they may have not. This is clinically relevant as it may produce instability. This was similarly found for COR offset, with errors as high as 8mm and 4.2mm for novice and experienced surgeon groups, respectively. Modern RSA systems allow for offset options in multiple planes, including the superior-inferior plane; however, errors of up to 8 mm are greater than most system recommendations and may result in clinically relevant notching.

Our measurement criteria were adopted from previously published methods for version, inclination, and offset.^15^^,^^21^^,^^29^^,^^35^ Rotational assessment of glenoid morphology has been previously described in native scapula and we adapted this methodology, defining the augment situated in the defect completely as 0° and as the reference.^5^ Many methods of glenoid inclination measurements have been published for native glenoids, and we used the method of the beta angle which has also previously been shown to have excellent inter-rater reliability for RSA implanted glenoids.^41^ The use of 3D CT DRRs has been performed on both native and operated glenoids including RSA.^6^^,^^22^^,^^39^^,^^40^ This method of assessment has previously shown high inter-rater reliability as we have shown in our study. This method of assessment has also been shown to have excellent correlation to, and agreement with, standard 3D CT measurements and as such, we used this method as our reference value, or ground truth for comparison.

Limitations of the study include small sample size (n = 8). To account for the small sample size, we used nonparametric testing and CIs. Superior glenoid defects were created using a standardized jig, which may have resulted in subtle variations in the imparted wear pattern location and size. This wear pattern will also look different than true wear patterns in clinical practice and may have been disorienting during implantation by novice surgeons and assessment of implant position by both groups of surgeons. Cadaver positioning (ie, limb stabilization angle) and retractor placement, although standardized, may have produced subtle variation in orientation between assessors. Preoperative CT scans were not collected to compare. Using 3D CT scans requires registration of the dataset on the CT scan and may lead to errors in measurement. Similarly, the use of 3D CT DRR may provide more avenues for erroneous measurement through interpretation of measurements and orientation of each view, although views were produced relative to fixed scapular landmarks. The use of 3D CT DRRs has not been explicitly validated for use in augmented glenoid RSA, although has previously been used to analyze standard glenoid baseplates.^41^ Interestingly, it may be that both novice and experienced surgeons refer to the glenoid enface plane during intraoperative assessment due to visualization through a deltopectoral approach as opposed to commercially available preoperative planning modalities that reference the scapula. This difference in perception could account for the variability seen between CT measurements and in-person, unassisted evaluation. Other studies have shown variations in implant positioning with large ranges similar to ours. Gregory et al showed that TSA had large variations in version (8 ± 9 [range: 17°-32°]), inclination (12 ± 13 [range: 21-50]), rotation (7 ± 14 [range: 30-46]), and offset (4 ± 3 [range: 7-15 mm]) in 68 patients when assessed by 3D CT.^18^ Boileau et al has previously described that superior wear patterns, particularly the concentrically eroded type E1 glenoid, may predispose the surgeon to placing the glenoid baseplate in superior inclination.^3^ Our results corroborate this and add that this may also be the case for E2 type wear patterns. Understanding intraoperative surgeon referencing and more challenging glenoid wear patterns is important to understand and characterize to mitigate glenoid implantation errors.

Both novice and experienced surgeons demonstrated clinically relevant bias in their intraoperative assessment of augmented glenoid baseplate orientation. The novice group had greater accuracy and less bias in measurement compared to the experienced group in all parameters. This can theoretically be explained by kinesthetic involvement of the novice surgeons, having just performed the guide pin placement and thus having more kinesthetic reference than the experienced group. The novice group also had time to visualize the orientation/size of the glenoid defect prior to and during implant insertion. Surgical tasks are learned through visual, auditory, and kinesthetic experience, and are best accomplished with all occurring simultaneously.^13^ Guided kinesthetic input over time encourages trainees to replicate the actions of an expert, and through practice, relying more on kinesthetic memory in situations where visual feedback is limited. Reeve et al evaluated various conditions of kinesthetic and visual input on errors in motor tasks, showing that participants predominantly focused on visual feedback over kinesthetic.^33^ Pinzon et al evaluated 20 participants performing kinesthetic tasks without motor or auditory input. Participants were able to recall orientation and direction through muscle memory, but were inaccurate of specific length measurements without visual cues.^31^ These studies provide insight into our results. Performing a shoulder arthroplasty without a priori understanding of the glenoid anatomy/wear pattern and limited visual cues of scapular anatomy can reduce performance. The addition of kinesthetic cues through palpation of the glenoid vault or scapular landmarks may assist in understanding of the anatomy and orientation of the glenoid. The experienced surgeon group may have had more inaccurate reported measurements because of this lack of kinesthetic input, relying on limited visual cues after the implant was inserted. This has implications for intraoperative teaching and learning curves for novice surgeons. If accurate orientation cannot be reliably achieved by the novice surgeons, then even with an experienced surgeon overseeing and guiding their performance, significant errors remain possible through visual assessment alone. Novice surgeons and trainees perform less critical implantation steps and may benefit from preoperative kinesthetic training such as cadavers or simulation (bone models or immersive virtual reality).^10^ Clinical outcome and radiographic correlation studies are required to determine the accuracy and effects of inaccurate placement of augmented baseplates in Favard CTA wear patterns.

## Conclusion

Intraoperative visual assessment of augmented glenoid baseplates in RSA for Favard E2 superior wear patterns is inaccurate for both novice and experienced surgeons. Assessment error is present for both novice and experienced surgeons in measured version, inclination, augment rotation, and superoinferior COR offset.

## Disclaimers

Funding: This work was supported by the Canadian Shoulder and Elbow Society (CSES).

Conflicts of interest: George Athwal reports personal fees from CONMED Linvatec (IP royalties), personal fees from Exactech, Inc. (IP royalties), personal fees from Parvizi Surgical Innovation (stock/stock options), personal fees from PrecisionOS (stock/stock options), personal fees from Reach Orthopedics (stock/stock options), personal fees from Stryker as a paid consultant and provision of research support (IP royalties), and personal fees from Wright Medical Technology (IP royalties). Dr. Athwal also discloses that he is a member of the editorial/governing board for JSES. Danny Goel discloses a conflict of interest with PrecisionOS as the founder and employee and thus receives personal fees (stock/stock options); outside of this, there are no further conflicts to disclose related to the subject of this article. The other authors, their immediate families, and any research foundation with which they are affiliated have not received any financial payments or other benefits from any commercial entity related to the subject of this article.
